# Design and Optimization of NR-Based Stretchable Conductive Composites Filled with MoSi_2_ Nanoparticles and MWCNTs: Perspectives from Experimental Characterization and Molecular Dynamics Simulations

**DOI:** 10.3390/polym16111444

**Published:** 2024-05-21

**Authors:** Ruifeng Jiang, Yanbin Ma, Zhuojun Fan, Yongping Chen, Tingting Zheng, Rentong Yu, Jianhe Liao

**Affiliations:** 1 School of Materials Science and Engineering, Hainan University, Haikou 570228, China; m13681005389@163.com (R.J.); yanbin.ma@hainanu.edu.cn (Y.M.); chenyp@hainanu.edu.cn (Y.C.); 2 School of Mathematics and Statistics, Hainan University, Haikou 570228, China; 15211041348@163.com; 3 School of Science, Qiongtai Normal University, Haikou 571127, China; qiaolezi621@163.com

**Keywords:** electrical conductivity composites, natural rubber, molecular dynamic simulation

## Abstract

Stretchable conductive composites play a pivotal role in the development of personalized electronic devices, electronic skins, and artificial implant devices. This article explores the fabrication and characterization of stretchable composites based on natural rubber (NR) filled with molybdenum disilicide (MoSi_2_) nanoparticles and multi-walled carbon nanotubes (MWCNTs). Experimental characterization and molecular dynamics (MD) simulations are employed to investigate the static and dynamic properties of the composites, including morphology, glass transition temperature (*T*_g_), electrical conductivity, and mechanical behavior. Results show that the addition of MoSi_2_ nanoparticles enhances the dispersion of MWCNTs within the NR matrix, optimizing the formation of a conductive network. Dynamic mechanical analysis (DMA) confirms the *T*_g_ reduction with the addition of MWCNTs and the influence of MoSi_2_ content on *T*_g_. Mechanical testing reveals that the tensile strength increases with MoSi_2_ content, with an optimal ratio of 4:1 MoSi_2_:MWCNTs. Electrical conductivity measurements demonstrate that the MoSi_2_/MWCNTs/NR composites exhibit enhanced conductivity, reaching optimal values at specific filler ratios. MD simulations further support experimental findings, highlighting the role of MoSi_2_ in improving dispersion and mechanical properties. Overall, the study elucidates the synergistic effects of nanoparticles and nanotubes in enhancing the properties of stretchable conductive composites.

## 1. Introduction

Although traditional rigid electronic products dominate the consumer electronics market, over the past decade, stretchable electronics have become a crucial market segment, particularly for personalized electronic devices, electronic skins [1,2,3,4], artificial implant devices [5,6,7,8], lightweight mobile electronic devices [9,10,11], and so on. Stretchable conductive composites are typically fabricated via two methods [12]. The first entails embedding non-ductile conductive materials, such as metal wires, into an elastic matrix; these originally rigid materials will be designed into a buckled structure to obtain stretchability [13,14]. Alternatively, a thin conductive film consisting of metal, carbon nanotubes, or graphene can be deposited on an elastomer surface; electronic conductivity, stretchability, and transparency can be improved by this elastomer–film structure [15,16].

Second, conductive fillers can be introduced to the insulating elastic matrix to achieve nanocomposites. Nanoscale conductive fillers are typically classified into zero-dimensional nanoparticles [17,18], one-dimensional nanowires/nanotubes [19,20,21], and two-dimensional nanoflakes [22,23]. For the purpose of low production cost and outstanding mechanical properties, zero-dimensional nanoparticles, such as carbon black, Au nanoparticles, and Ag nanoparticles, have been adopted

Carbon nanotubes (CNTs) and silver nanowires are extensively employed in stretchable conductive composites, owing to their high aspect ratios [12,14,15]. However, the conductivity associated with percolation is highly strain-sensitive, declining sharply under high strain and then undermining their cycle stability. Nevertheless, nanoparticles can result in the integrity of the conductive network under high-strain conditions.

The selection of a suitable flexible substrate is crucial to fabricate stretchable conductive materials. Polydimethylsiloxane (PDMS) has been predominantly used as a substrate material for stretchable electronic devices, owing to its stable chemical properties, high thermal stability, optical transparency, and biocompatibility [24,25,26,27]. However, its inherent low surface energy reduces adhesion and interfacial bonding strength between conductive fillers and the PDMS matrix and also reduces elongation at break for PDMS. Consequently, the inter-phase of the composite would deteriorate under high strain or repeated stretching, having a negative effect on the performance of the stretchable electronic devices [28]. As a renewable bio-synthetic polymer in comparison to fossil resources, natural rubber (NR) agrees with the principle of eco-friendly and sustainable development. Moreover, its inherent chain flexibility and ultra-high molecular weight endow it with superior resilience under prolonged strain conditions [29,30,31,32]. In addition, the NR processing methodology facilitates the continuous production of large-area stretchable conductive films utilizing NR latex as the primary raw material.

Among the conductive fillers, carbon nanotubes, which are recognized as a novel carbon-based material, exhibit remarkable electrical and mechanical properties, characterized by a high aspect ratio and conductivity. Consequently, they facilitate the formation of a conductive network in the polymeric matrix composite. Notable enhancements in the electromagnetic shielding effectiveness and mechanical properties of polymer composites can be achieved with a marginal increase in the content of these carbon-based materials [33,34,35].

In general, carbon nanotubes can be synthesized via three different routines, i.e., electric arc discharge (Arc-Discharge) [36,37,38], laser ablation [39,40,41], and chemical vapor deposition (CVD) methods, and the microwave irradiation method [42,43,44,45,46]. Of them, the arc discharge method is characteristic of scaled production and cost-efficiency. Nevertheless, the raw CNTs necessitate further purification, and the CNTs are hard to be characterized explicitly. In contrast, high-level CNTs can be produced at room temperature by the laser ablation method. Raid et al. [39] reported the preparation of high-level CNTs without purification. However, CNTs cannot be synthesized on a large scale by this mean. Fortunately, high-grade CNTs can be achieved with the advantages of high efficiency, continuous production, and low production cost [47,48]. Nowadays, the CVD method has been widely approached in the laboratory and in industrial production [49]. In addition, as a novel and cost-efficient method to synthesize CNTs, microwave irradiation is advantageous, due to its ability to provide a rapid and uniform heating rate, which can be selectively directed to a specific area [42,43,44]. Meanwhile, rather than the majority of the existing methods, which is using carbon in the form of graphite, methane, ethylene, or acetylene as the starting material, microwave irradiation can provide a more plentiful choice of carbon sources, such as coal [45] and biochar [46].

Moreover, the incorporation of multidimensional fillers, including zero-dimensional nanoparticles and one-dimensional nanotubes, elicits a synergistic effect, further augmenting the mechanical and electrical properties of the composites [50,51,52]. Some conductive nanoparticles, such as Ag [53], Ni [54], and Cu [52], have been approached in many researches of stretchable conductive materials. However, metal filler will be corroded with oxygen on account of the high oxygen diffusion coefficient in the elastomer. molybdenum disilicide (MoSi_2_) exhibits an excellent high-temperature oxidation resistance and low electrical resistivity (about 15 μΩcm at room temperature) [55]. In addition, MoSi_2_ has been widely used to reinforced polymer composites. Zhang et al. [56] reported a carbon fiber/boron phenolic resin composite modified by MoSi_2_ and mica with high mechanical, thermal, and ablation properties.

Although some measurement methods, such as in-situ transmission electron microscopy (in-situ TEM) and atomic force microscope (AFM), can be employed to clearly observe the dynamic process of materials at the atomic scale, the stability of the sample during the testing process and the difficulty of ultrafast imaging at the nano-second scale limit the widespread application of these in-situ imaging technologies [57,58,59]. Fortunately, at an atomic scale, molecular dynamics (MD) simulations provide distinct advantages in investigating both static and dynamic properties of polymer composites, compared with conventional experimental approaches [60]. Moreover, micro-structure evolution can be revealed in detail. In this way, complex models and ideal well-dispersed systems can be created [61]. MD simulations facilitate visualization together with quantitative analysis in a dynamic process at the micro-scale. In the past 30 years, extensive research on polymer materials [62,63,64,65,66,67,68,69] and conductive composites [70,71,72,73] have been conducted using MD simulations, owing to the rapid development of computer technology. MD simulation can measure and statistically analyze the glass transition [62,63], mechanical properties [64,65], rheological properties [66,67], and microscopic dynamic structure [68,69] of polymers and their composite materials. In addition to the method mentioned above, the statistical conductive network model developed by Fang et al. [74] was adopted to calculate the change in conductive pathways in the tensile process of MD simulations in this research.

In this study, we focused on NR-based composites containing MoSi_2_ nanoparticles and multi-walled carbon nanotubes (MWCNTs) and studied the effects of nanoparticles/nanotubes on the properties of the composites through morphology analysis, glass transition temperature analysis, mechanical testing, and electrical conductivity testing. Moreover, coarse-grained MD (CGMD) simulations employing an all-atom force field fitting were conducted to construct six sets of models for different filler mass ratios (m(MoSi_2_):m(MWCNTs) = 5:0, 4:1, 3:2, 2:3, 1:4, 0:5). Subsequently, the glass transition temperature (*T*_g_) and radial distribution function (RDF) were calculated. Simulated tensile testing was performed based on the state of equilibration, followed by an analysis of the conductive network change in the simulation of the tensile process.

## 2. Materials and Methods

### 2.1. Preparation of the NR Vulcanizates

NR with the grade V was supplied by the Jinfu Plant of Hainan Rubber Industry Group Co., Ltd., Haikou, China. The 97% multi-walled carbon nanotubes (MWCNTs) with an inner diameter of 3–5 nm, outer diameter of 8–15 nm, and specific surface area ≥ 250 m^2^/g and that were synthesized by the CVD method were purchased from Shenzhen Guosen Pilot Technology Co., Ltd., Shenzhen, China. MoSi_2_ with the size of 1000 nm was purchased from Suzhou Yuante New Material Co., Ltd., Suzhou, China. NR was masticated and then cured according to ISO 1658:2015 [75] and GB/T 15340-2008 [76]. The vulcanizates formulation is shown in Table S1. MoSi_2_ and MWCNTs were compounded as fillers to prepare the MoSi_2_/NR, MWCNTs/NR, and MoSi_2_/MWCNTs/NR composites. The conductive composites formulations are shown in Table S2. After mixing for 2–4 h at room temperature (about 25 °C), 4–6 g of the compound was taken and placed in a moving die rheometer for testing. The test temperature was 145 °C, and the test time was approximately 60 min. Finally, the vulcanization was performed using a flat vulcanization instrument. The vulcanization temperature was 145 °C, the vulcanization time was the optimum vulcanization time (*t*_90_), and the vulcanization template thickness was 2 mm. The thickness of the sample was kept at 2 mm.

### 2.2. Molecular Dynamics Simulations

All of the non-bonded interactions were modeled using the expanded truncated and shifted Lennard–Jones potential (LJ potential):

(1) $$E=\left\{\begin{array}{ll} 4\varepsilon \left[{\left(\frac{\sigma }{r-\Delta }\right)}^{12}-{\left(\frac{\sigma }{r-\Delta }\right)}^{6}\right] & r<r_{cutoff}+\Delta \\ 0 & r\geq r_{cutoff}+\Delta \end{array}\right.$$

where *ε* represents the pair interaction energy parameter; *r* represents the distance between each two interaction sites; ∆ accounts for the effect of the excluded volumes of different interaction sites, representing the rigid volume of coarse particles; and *r_cutoff_* represents the pair cut-off distance at which the LJ potential is truncated and shifted so that the energy is equal to zero at this distance.

The interactions between adjacent bonded beads and three consecutive beads were modeled using the harmonic potential formula and illustrated in Equations (2) and (3), respectively:

(2) $$E_{bond}=K_{bond}{\left(r-r_{0}\right)}^{2}$$

(3) $$E_{angle}=K_{angle}{\left(\theta -\theta_{0}\right)}^{2}$$

where *r*_0_ is the equilibrium bond distance, and *K_bond_* and *K_angle_* represent the strength factors.

The coarse-grained force field used for the MoSi_2_/MWCNTs/NR systems was attained using the VOTCA software (v2023) [77,78,79] based on the OPLS–AA force field [80] via the iterative Boltzmann inversion (IBI) [81]. The force field parameters are shown in Table 1, and the criterion to construct the coarse-grained model is presented in Table S3. The bead number of the polyisoprene (PI) chain for each system was 1000, the bead number of the MWCNTs chain was 159, and the mass ratio of MoSi_2_ and MWCNTs was based on the amount of the MoSi_2_/MWCNT components listed in Table S2.

All the MD simulations were conducted using the large-scale atomic/molecular massively parallel simulator (LAMMPS) software (lammps-8Feb2023) developed by Sandia National Laboratories [82]. Throughout the MD simulations, the following settings were used: the NPT ensemble was executed for all the systems, the velocity Verlet algorithm was employed to integrate the equations of motion with a time step of 0.001 *τ*, and the Nose–Hoover thermostat, as well as barostat and periodic boundary conditions, was used in all three directions.

In the balancing process, we used the method proposed by Auhl et al. [83] to model the well-equilibrated melts of long-chain polymers. The systems were run for 10,000 *τ,* with a time step of 0.001 *τ*, and P* = 1.0; T* was oscillated from 1.0 to 5.0.

After the balancing process, all the equilibrium structure data were copied into two groups: One was the trajectory data continuously simulated for 1000 *τ* with T* = 1.0 and P* = 1.0, including static and dynamic structural information in the equilibrium state. These trajectory data will be discussed in the interphase topology analysis. The other group, based on the free volume theory, started from a gradient cooling simulation process with a T* from 5.0 to 0.04 for 36,000 *τ*. The cooling process was divided into two parts: First, temperature was decreased from 5.0 to 1.0 in 25 loops, each loop consisting of a decreasing temperature process with 400 *τ* and a constant temperature process with 400 *τ*. Second, temperature was decreased from 1.0 to 0.04 in 32 loops, each loop consisting of a decreasing temperature process with 250 *τ* and a constant temperature process with 250 *τ*. As a result, the glass transition temperature (*T*_g_) of each system was determined by identifying the temperature at which the specific volume changed from a rubber state to a glass state. The *T*_g_ was indicated by the highest point of the second derivative curve calculated from the temperature–volume curve.

Mechanical properties such as tensile stress were computed by applying uniaxial deformation via the SLLOD equations of motion [84]. The tensile strain rate was set to be 0.001/*τ*, which was believed to be sufficiently slow to approach the limiting behavior for the equilibrium deformation process. The volume of the simulation box was maintained at a constant during the deformation process.

### 2.3. Characterization

#### 2.3.1. Static Mechanical Properties

According to GB/T 528-1998 [85], the stress–strain curves of the samples were measured using a tensile testing machine (AL-7000-SU2, Qingdao Kangping High-speed Railway Technology Co., Ltd., Qingdao, China) with a strain rate of 500 mm/min at 25 °C. The samples were prepared to be a standard dumbbell-shaped sheet.

#### 2.3.2. Dynamic Thermomechanical Performance

Dynamic mechanical analysis (DMA; TA Q800, TA Instruments, New Castle, DE, USA) was performed to obtain the curve of tan *δ* as a function of temperature in a nitrogen atmosphere. DMA measurements were conducted at a heating rate of 3 °C/min and a frequency of 1 Hz. The temperature range was −100 °C–100 °C. In all cases, a preload of 0.01 N was applied.

#### 2.3.3. Differential Scanning Calorimetry (DSC) Measurements

*T*_g_ was measured using a Q2000 (TA Instruments, New Castle, DE, USA) differential scanning calorimeter. Under the protection of a nitrogen atmosphere, the samples were heated from −85 °C to 25 °C at a ramp rate of 10 °C/min. The specific *T*_g_ was measured by the differentiation method.

#### 2.3.4. Scanning Electron Microscopy (SEM)

The surface morphology of different NR-based composites also observed was observed with a ZEISS Sigma 300 scanning electron microscope (Oberkochen, Germany), which was made in Germany. The vulcanized rubber was cut into a thin sheet with uniform thickness and then adhered to the conductive rubber. After spraying gold, the morphologies of composites were observed with an acceleration voltage of 5 kV.

#### 2.3.5. Conductivity Measurements

The resistance of composite films was measured using a digital multimeter (Tektronix DMM6500, Beaverton, OR, USA). Electrical conductivity was calculated according to the following equation:

(4) $$\sigma =\frac{L}{SR}$$

where ***L*** is the length of the test sample, ***S*** is the cross-sectional area of the sample, and ***R*** is the conductive resistance of the sample.

## 3. Results

### 3.1. Morphology of Different NR-Based Composites

**Figure 1:**
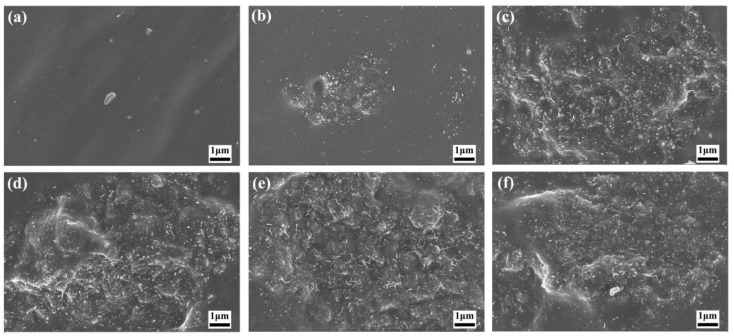
SEM images of the MWCNTs/NR composites with different MWCNT loadings: (**a**) 0 phr, which is the control group; (**b**) 1 phr; (**c**) 2 phr; (**d**) 3 phr; (**e**) 4 phr; and (**f**) 5 phr.

**Figure 2:**
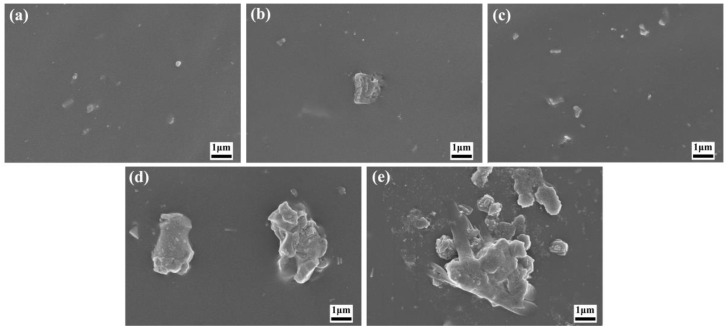
SEM images of the MoSi_2_/NR composites: (**a**) 1 phr of MoSi_2_; (**b**) 2 phr of MoSi_2_; (**c**) 3 phr of MoSi_2_; (**d**) 4 phr of MoSi_2_; and (**e**) 5 phr of MoSi_2_.

**Figure 3:**
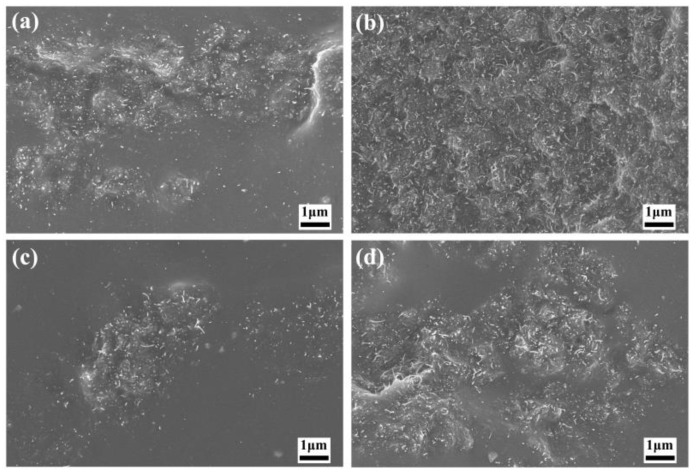
SEM images of the MoSi_2_/MWCNTs/NR composites with MWCNTs/MoSi_2_ mass ratios: (**a**) 1:4; (**b**) 2:3; (**c**) 3:2; and (**d**) 4:1.

Figure S1 shows the SEM image of pure filler, including MoSi_2_ and MWCNTs. Figure 1 shows the SEM image of the MWCNTs/NR system. When the MWCNT filler content ranged from 1 to 3 phr, a uniform dispersion within the NR matrix was achieved. However, when increasing the filler content, the agglomeration of MWCNTs can be observed. As a consequence, the uniform dispersion of MWCNTs in the NR matrix cannot be achieved, which would give rise to the deterioration of the mechanical properties of the MWCNTs/NR composite. Figure 2 depicts the SEM image of the nanoparticles/NR composites (MoSi_2_/NR), exhibiting a dispersion pattern of the fillers in the matrix, which is consistent with that of the MWCNT filler, as the added amount varies. Notably, at the filler content of 4 phr or 5 phr, nanoparticle aggregation can be observed. As shown in Figure 3, the addition of MoSi_2_ effectively enhanced the dispersion of MWCNTs while maintaining the total filler fraction. Particularly, at a MoSi_2_/MWCNTs ratio of 2:3, the filler dispersion within the NR matrix was optimized. This observation supports the synergistic effect of nanoparticles and nanotubes in promoting the formation of a conductive network within the matrix, thereby enhancing its electrical conductivity.

**Figure 4:**
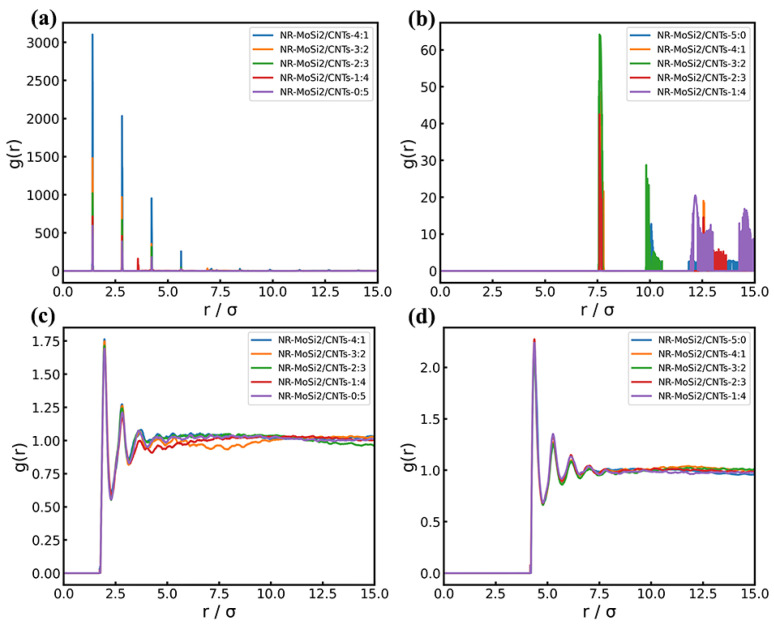
Radial distribution function (RDF) of MoSi_2_/MWCNTs/NR systems between (**a**) MWCNT beads and MWCNT beads; (**b**) MoSi_2_ beads and MoSi_2_ beads; (**c**) MWCNT beads and NR beads; and (**d**) MoSi_2_ beads and NR beads.

Furthermore, the radius distribution function (RDF) of the MoSi_2_/MWCNTs/NR system was calculated by means of MD and depicted in Figure 4. Figure 4a,b illustrates the dispersion of the two fillers (MoSi_2_ and MWCNTs), respectively. It can be found that the dispersion of fillers in the NR matrix was perfect, even without surface modification. Figure 4c,d displays the radius distribution between the MWCNTs/NR and MoSi_2_/NR, respectively. The first peak presented in the RDF results (*r* = 2.07 in Figure 4c, *r* = 4.5 in Figure 4d) both agreed with the ideal distance between NR and fillers based on force field parameters, indicating that a well-balance state was achieved.

### 3.2. Analysis of Glass Transition

**Figure 5:**
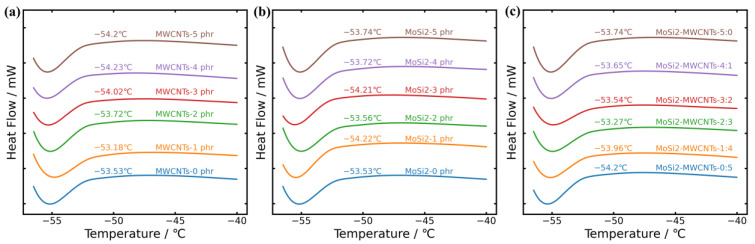
DSC curves of the composites: (**a**) MWCNTs/NR; (**b**) MoSi_2_/NR; (**c**) MoSi_2_/MWCNTs/NR.

Figure 5a illustrates the variation in *T*_g_ for the MWCNTs/NR system. Upon the addition of 2 to 3 phr of MWCNTs, a slight increase in *T*_g_ can be observed, relative to the *T*_g_, which MWCNTs added up to 1 phr. However, the overall trend revealed a gradual decrease in *T*_g_ (from −53.53 °C to −54.2 °C) as the filler concentration increased. This phenomenon may be attributed to the hollow structure of MWCNTs, which provides additional free volume, enhancing the molecular chain mobility. Figure 5b depicts the *T*_g_ variation for the MoSi_2_/NR system. With increasing filler content, *T*_g_ underwent a slight decline, followed by an increasing trend, suggesting that a limited quantity of filler particles enhanced the chain segment mobility at low temperatures. In addition, as shown in Figure 5c, the incorporation of MoSi_2_ dominated the increasing trend of *T*_g_ in MoSi_2_/MWCNTs/NR systems. Hence, excessive MoSi_2_ content is adverse to keeping a low *T*_g_ in composites.

**Figure 6:**
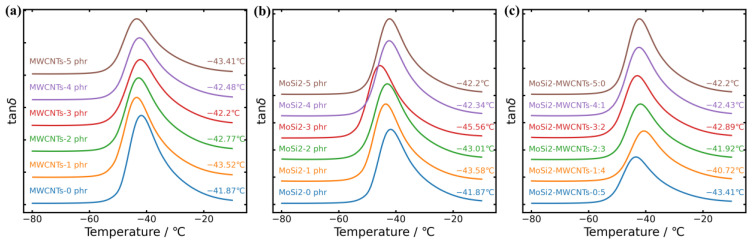
DMA curves of composites: (**a**)MWCNTs/NR; (**b**) MoSi_2_/NR; (**c**) MoSi_2_/MWCNTs/NR.

**Figure 7:**
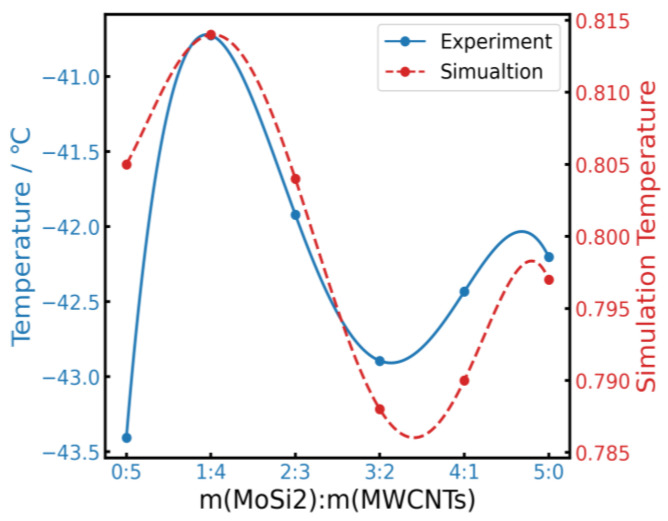
Comparison of *T*_g_ values obtained from DMA measurements (blue solid line) and simulated (red dashed line) *T*_g_ values.

To further understand the reinforcement mechanism of the nanoparticles/nanotubes within the NR matrix, dynamic mechanical tests were conducted from −80 °C to 0 °C. Figure 6 illustrates the relationship of the loss angle tan *δ* and the fillers content in MWCNTs/NR, MoSi_2_NR, and MoSi_2_/MWCNTs/NR composites (Figure 6a, Figure 6b, and Figure 6c, respectively), with the temperature spanning from −80 °C to 0 °C. Notably, α transition temperature was found to be a decreasing trend with the increase in MWCNTs content, which agrees with the results of DSC, indicating a reduction in *T*_g_, which agrees with the results of DSC. A similar trend was observed for the MoSi_2_/NR system, where the lowest *T*_g_ was observed for the composite containing 3 phr of MoSi_2_, followed by a continuous increase in *T*_g_ as the filler fraction increased to 5 phr. Within the MoSi_2_/MWCNTs/NR system, the addition of 1 phr of MoSi_2_ considerably increased *T*_g_, which then decreased until the mass fraction of MoSi_2_ reached 3 phr. Subsequently, when the increase in the MoSi_2_ fraction reached 5 phr, *T*_g_ increased gradually. Moreover, as depicted in Figure 7, *T*_g_ variations measured for the MoSi_2_/MWCNTs/NR system measured via DMA closely aligned with those obtained from MD simulations, as indicated by the red dashed line. This concordance highlights the capability of MoSi_2_ to enhance system dispersion; however, excessive MoSi_2_ filler loading is not beneficial. The lowest *T*_g_ was achieved for the MoSi_2_/MWCNTs/NR system with an approximately 3 phr MoSi_2_ addition. To elucidate the influence of MoSi_2_/MWCNTs on *T*_g_ in depth, MD simulation was performed, and the *T*_g_s of the composites are shown in Figure 7. By comparing the results of the simulation and DMA, a similar relationship between the mass fraction of fillers and *T*_g_ can be ascertained. *T*_g_ showed a rapid increase when MoSi_2_ attained 1 phr. Then, *T*_g_ continually decreased to the lowest point when MoSi_2_ added up to 3 phr. This phenomenon implies that the content of MoSi_2_ could dominate the change in *T*_g_ in MoSi_2_/MWCNTs/NR systems.

### 3.3. Mechanical Properties

**Figure 8:**
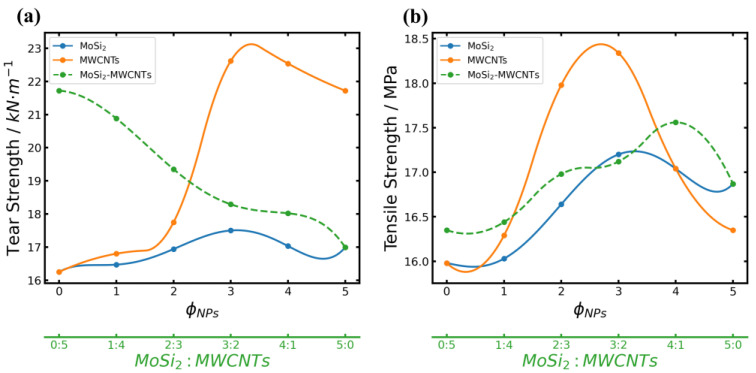
Mechanical properties of the inorganic fillers/NR composites: (**a**) tear strength and (**b**) tensile strength of MoSi_2_/NR (blue solid line), MWCNTs/NR (orange solid line), and MoSi_2_/MWCNTs/NR (green dashed line).

The mechanical properties of the composites were evaluated using a universal tensile tester. Figure 8 illustrates the tear strength and tensile strength of the MoSi_2_/NR, the MWCNTs/NR, and the MoSi_2_/MWCNTs/NR systems. In Figure 8a, there is almost no enhancement of tear strength with just the incorporation of MoSi_2_ in composites, as depicted by the blue curve. Conversely, the MWCNTs/NR that included highly linear one-dimensional MWCNTs (orange curve) notably improved tear resistance; tear strength was remarkedly enhanced, especially when 2–3 phr of MWCNTs was added. Furthermore, when MWCNTs added up to a mass fraction of 5 phr, a downward trend was observed; it is possibly attributed to poor compatibility between the MWCNTs and NR. These characteristics can be observed in MoSi_2_/MWCNTs/NR systems. With the decrease in MWCNTs and increase in MoSi_2_, tear strength showed a continuous downward trend. Thus, MoSi_2_ has a negative influence on the tear strength of composites. MoSi_2_ has a negative influence on the tear strength of composites.

Figure 8b illustrates the variation in tensile strength. Although the MWCNTs/NR system demonstrated remarkable tensile resistance, displaying the best tensile strength performance at a mass fraction equal to 3 phr, its tensile strength was eventually surpassed by MoSi_2_ at higher mass fractions (4–5 phr). However, the MoSi_2_/MWCNTs/NR system exhibited a progressive increase in tensile strength, outperforming the former two systems at a mass fraction of 4 phr, possibly owing to synergistic effects. Spherical MoSi_2_ particles effectively inhibited the formation of cracks perpendicular to the tensile direction, enhancing the overall tensile strength of the composite.

**Figure 9:**
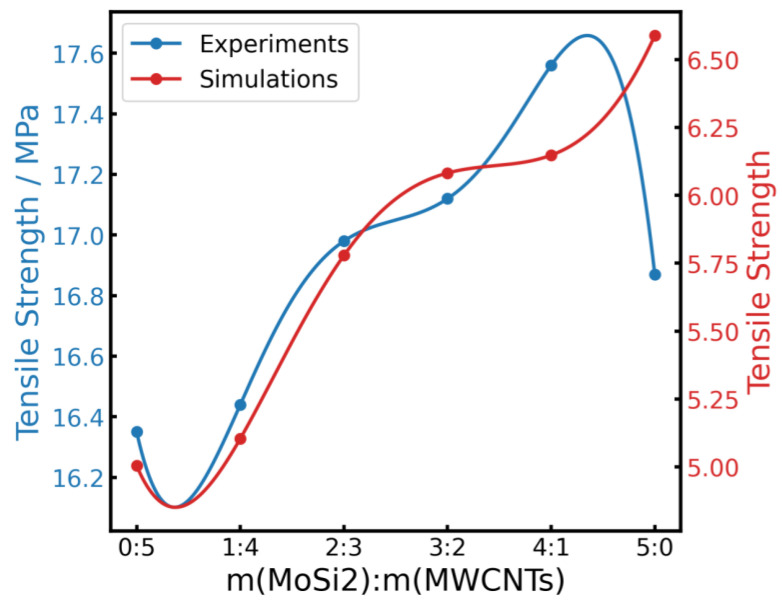
Comparison of the tensile strengths obtained from experiments (blue line) and the simulated (red line) tensile strengths.

The results of MD simulations further support the findings depicted in Figure 8. As shown in Figure 9, the mechanical properties of the MoSi_2_/MWCNTs/NR system obtained using MD simulations are presented with those of mechanical testing. The tensile strength increased with increasing MoSi_2_ filler quantities across the first five mass ratios (the m(MoSi_2_): m(MWCNTs) ratio equal to 0:5, 1:4, 2:3, 3:2, 4:1), yielding the best mechanical property when the ratio of the two reached 4:1. However, the tensile strength of the composite containing MoSi_2_/MWCNTs resulting from MD simulation was much higher than that measured by experimental tensile testing when the mass ratio of MoSi_2_/MWCNTs was 5:0. This can be explained by the fact that MD simulations are always performed in a small scale of time and space; in order to obtain simulation results in a limited computational time, the tensile simulation will be performed at a very high tensile rate. Compared with the experimental tensile rate, it may extend the increasing trend of the tensile strength in the MD simulation result. Furthermore, the simulation models of composites will have a more ideal structure than experimental composites. Thus, the tensile strength of the simulation was still increasing when the mass ratio of MoSi_2_/MWCNTs was 5:0.

### 3.4. Electrically Conducting Properties

**Figure 10:**
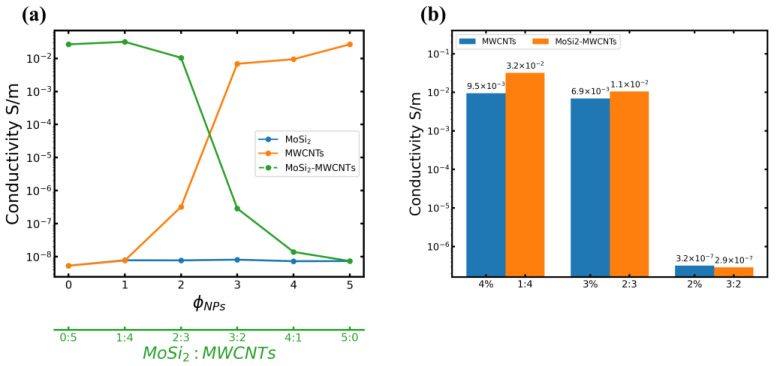
(**a**) Conductivities of NR–MoSi_2_ (blue solid line), MWCNTs/NR (orange solid line), and MoSi_2_/MWCNTs/NR (green dashed line); (**b**) conductivity bar plots ofMWCNTs/NR (blue) and MoSi_2_/MWCNTs/NR (orange).

Electrical conductivity serves as a key parameter that reflects materials’ ability to conduct electric current. The results of conductivity tests for all the systems are presented in Figure 10. As shown in Figure 10, the utilization of MWCNTs as conductive fillers increased the conductivity of MWCNTs/NR composites concomitant with filler content augmentation, indicating the formation of a conductive network structure within NR. With the incorporation of 3 phr of MWCNTs, the composite conductivity exceeded that of the matrix (control sample) by 6 orders of magnitude, indicating that the 2–3 phr filler content met with the formation of the three-dimensional conductive network, i.e., the 2–3 phr filler was higher than the percolation threshold in the NR-based composite.

By altering the filler composition, a blend of zero-dimensional nanoparticles and one-dimensional nanotubes was incorporated into the NR matrix. At a 5 phr MWCNTs content, MWCNTs/NR composites exhibited a conductivity of 2.67 × 10^−2^ S/m. Interestingly, for the MoSi_2_/MWCNTs/NR composites, the conductivity exhibited a slight increase after the addition of 1 phr of MoSi_2_ (i.e., with a MoSi_2_: MWCNTs mass ratio of 1:4), as depicted in Figure 10b. Furthermore, at a MoSi_2_: MWCNTs mass ratio of 2:3, high conductivity (1.05 × 10^−2^ S/m) was sustained, indicating that the introduction of an appropriate quantity of nanoparticles improves the dispersion of MWCNTs within the NR matrix, which facilitates the establishment of a relatively comprehensive free-electron transfer pathway and thus enhances material conductivity. Further, at a MoSi_2_: MWCNTs mass ratio of 2:3, the composite conductivity surpassed that of the MoSi_2_: MWCNTs mass ratio of 3:2 by 5 orders of magnitude, indicating that the percolation threshold of the MoSi_2_/MWCNTs/NR system occurs within the mass ratio range of 2:3 to 3:2. Furthermore, at a MoSi_2_: MWCNTs mass ratio of 1:4, MoSi_2_/MWCNTs/NR composites exhibited the highest conductivity.

**Figure 11:**
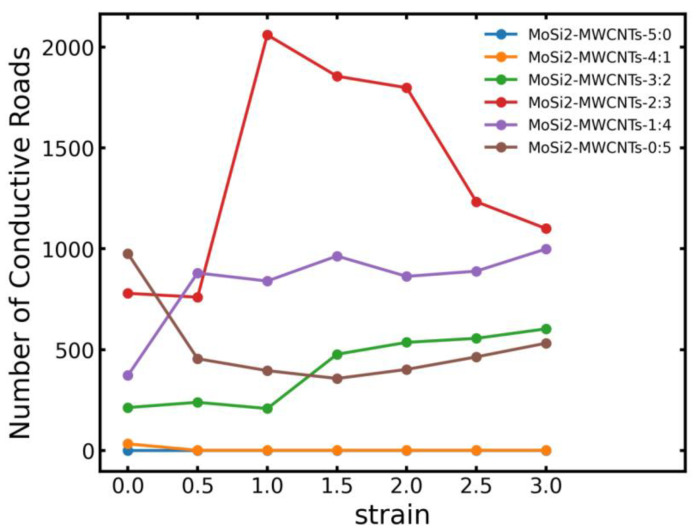
Number of conducting pathways of the model with varied strains in the *z*-axis direction (simulation).

According to the trajectory data derived during the stretching simulations, the number of conductive pathways along the stretching direction was calculated. The information of conductive pathways across different composite systems during stretching along the z-axis is shown in Figure 11. At zero strain, the conductive pathways of the MoSi_2_/MWCNTs/NR-5:0 and MoSi_2_/MWCNTs/NR-4:1 systems were insignificant (always 0). Upon reaching a CNT content of 2 phr, a substantial change in the number of conductive pathways was observed from approximately 0 to about 250. When the mass fraction of MWCNTs ranged from 2 phr to 3 phr, there was a significant increase in the conductive pathways number, implying that the percolation threshold of the MoSi_2_/MWCNTs/NR system under non-tensile conditions lies between 2 and 3 phr. Under tensile strain ranging from 0 to 300% along the z-axis, both the MoSi_2_/MWCNTs/NR-3:2 and MoSi_2_/MWCNTs/NR-1:4 systems exhibited an augmentation in the number of conductive pathways after stretching, followed by a plateau, indicative of the stretching-induced formation of conductive pathways within the system. At a strain of 50–100%, an obvious increase in the number of conductive pathways was observed in the MoSi_2_/MWCNTs/NR-2:3 system, followed by a subsequent decline in proximity to the number observed in the MoSi_2_/MWCNTs/NR-1:4 system.

This phenomenon may be ascribed to the fact that the 2:3 filler mass ratio of the MoSi_2_/MWCNTs was near the value of the percolation threshold of the composite system. In this sense, minor disturbances can amplify the formation process of the conductive network, remarkably increasing the number of conductive pathways. Furthermore, the pure CNT system exhibited a downward trend in the number of conductive pathways after stretching, indicating the synergistic effects of nanoparticles and MWCNTs in the stretching-induced orientation for constructing conductive pathways.

## 4. Conclusions

In summary, the investigation into NR-based stretchable composites filled with MoSi_2_ nanoparticles and MWCNTs provides valuable insights into the design and optimization of advanced materials for flexible electronics. The combination of experimental characterization and molecular dynamics simulations offers a comprehensive understanding of the structure–property relationships in these composites. A comparison of performance with other similar conductive composite is indicated in Table 2.

The morphological analysis reveals the importance of achieving uniform dispersion of fillers within the polymer matrix for optimal mechanical and electrical performance. Both MoSi_2_ nanoparticles and MWCNTs contribute synergistically to the formation of a conductive network, enhancing electrical conductivity and mechanical strength. However, careful consideration of filler content and ratio is necessary to avoid detrimental effects, such as agglomeration and decreased tear strength.

Furthermore, dynamic mechanical analysis and molecular dynamics simulations elucidate the impact of filler composition on the glass transition temperature, highlighting the role of MoSi_2_ in modulating the polymer chain mobility and enhancing the overall mechanical properties of the composites.

## Figures and Tables

**Table 1:** Coarse-graining process and force field parameters for IBI calculations.

	Atomic Force Field			Coarse-Graining Force Field		
	PI	MWCNTs	MoSi_2_	PI	MWCNTs	MoSi_2_
**ε** **(kJ/mol)**	0.75	9.89	0.84	1	13.20	1.12
**σ** **(nm)**	0.6	1.8825	0.43	1	3.14	0.72
***K_bond_*** **(kJ/mol/nm^2^)**	4029	36,353	-	1934	48,417	-
***r*_0_** **(nm)**	0.495	0.967	-	0.825	1.612	-
***K*** *_angle_* **(kJ/mol/rad^2^)**	11.96	39,201	-	9.8	52,268	-
***Θ*_0_** **(kJ/mol/degree^2^)**	135	180	-	135	180	-
**Mass** **(Ar)**	68	2304	50,480	1	33.8	742.35
∆	-	-	-	0	0	4

**Table 2:** Comparison with results of other authors about the NR-based conductive composites.

	Conductivity (S/m)	Tensile Strength (MPa)	Ref.
MWCNTs/NR	10^−4^	23.3	[86]
CNT-Ag/NR	10^−5^	-	[87]
Cu_NPs/NR	1.4 × 10^−2^	12.79	[88]
MWCNTs/MGNR	10^−2^	17.4	[89]
MoSi_2_/MWCNTs/NR	3.18 × 10^−2^	16.42	this work

## Data Availability

Data are contained within the article.

## Supplementary Materials

The following supporting information can be downloaded at: https://www.mdpi.com/article/10.3390/polym16111444/s1, Table S1. Rubber compound formulation; Table S2. Natural-rubber-based composite compound formulation; Table S3. All-atom and coarse-grain models of PI and CNT; Figure S1. SEM images of MoSi_2_ (a) and MWCNTs (b).
