# Supplementary material for: Design and Optimization of NR-Based Stretchable Conductive Composites Filled with MoSi2 Nanoparticles and MWCNTs: Perspectives from Experimental Characterization and Molecular Dynamics Simulations
Source: Polymers (Basel). 2024 May 21;16(11):1444. doi: 10.3390/polym16111444 (PMC11175021; doi:10.3390/polym16111444)
Supplement: Supplementary file 1 [file polymers-16-01444-s001.zip › polymers-3013346-supplementary.pdf]

Table S1 Rubber compound formulation

Ingredients	Parts per hundred rubber (phr)
Natural rubber	100
Stearic acid	0.5
ZnO	6
2-Mercaptobenzothiazole	0.5
Sulfur	3.5

Table S2 Natural-rubber-based composite compound formulation

Ingredients	Parts per hundred rubber (phr)
Natural rubber	100
Stearic acid	0.5
ZnO	6
2-Mercaptobenzothiazole	0.5
Sulfur	3.5
$MoSi_2$-1000nm	1, 2, 3, 4, 5
MWCNTs	1, 2, 3, 4, 5
$MoSi_2$/MWCNTs	1/4, 2/3, 3/2, 4/1

Table S3 All-atom and coarse-grain models of PI and CNT

	**Atomic model**	**Coarse-graining model**
Polyisoprene	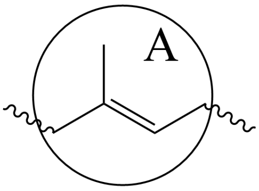	$\sim\sim A_1 — A_2 — A_3 \sim\sim$
CNT	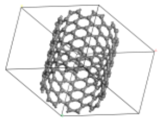	$\sim\sim B_1 — B_2 — B_3 \sim\sim$

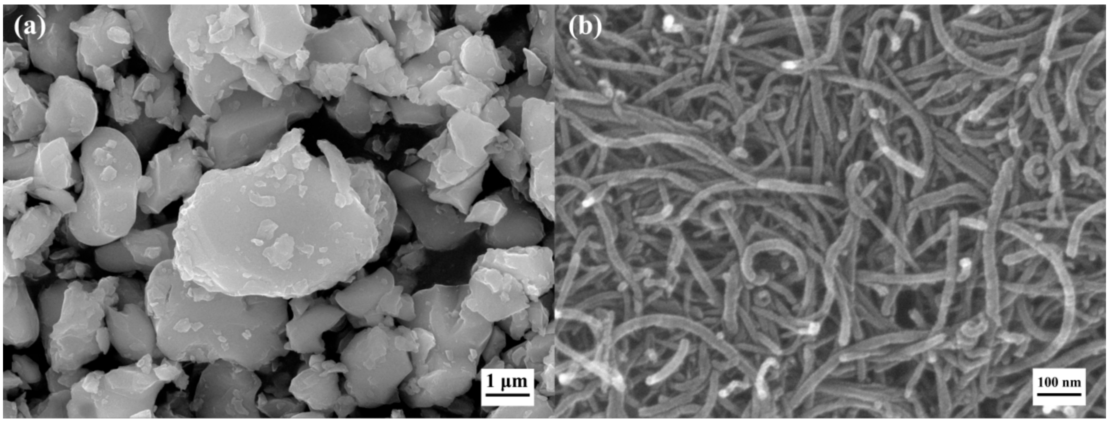

Figure S1 SEM images of $MoSi_2$ (a) and MWCNTs (b).
